# *MTHFR* Gene Variants and Major Depressive Disorder in Mexican-Mestizos

**DOI:** 10.3390/bs16091639

**Published:** 2026-09-14

**Authors:** Miriam Pavelth Casillas-Ávila, Christian Gabriel Toledo-Lozano, Luis Ángel Montes Almanza, Ramón Mauricio Coral-Vázquez, Isabel Ramírez Sixtos, Omar Medrano Espinosa, Silvia García, Cristina Rodríguez Hernández, Froylan Arturo García Martínez, Edgar Oswaldo Zamora González, Guillermo García Castillo, Benjamín Gómez Díaz, Matxil Violeta Díaz Benavides, Stephanie Alejandra Rosas-Maldonado, Cristina Gutiérrez Mendoza, Carlos Palma Flores, Luz Berenice López-Hernández

**Affiliations:** 1 Programa de Doctorado en Genética Humana, Universidad de Guadalajara, Guadalajara 44100, Mexico; mipavelth@hotmail.com; 2 Hospital Civil “Dr. Antonio González Guevara”, Tepic 63169, Mexico; 3 Subdirección de Enseñanza e Investigación, Centro Médico Nacional “20 de Noviembre”, ISSSTE, Mexico City 03100, Mexico; christian.toledo@issste.gob.mx (C.G.T.-L.); silvia.garcia@issste.gob.mx (S.G.); garciacastillog@yahoo.com.mx (G.G.C.); 4 Programa de Maestría en Ciencias de la Salud, Instituto Politécnico Nacional, Centro Médico Nacional “20 de Noviembre”, ISSSTE, Mexico City 03100, Mexico; luis.montes@condeinvestigacion.org (L.Á.M.A.); isamarisixtos1990@hotmail.com (I.R.S.); omarmed5@gmail.com (O.M.E.); contacto@drapsiqcristinarodriguez.com (C.R.H.); froylan.garcia@condeinvestigacion.org (F.A.G.M.); 5 Sección de Estudios de Posgrado e Investigación, Escuela Superior de Medicina, Instituto Politécnico Nacional, Mexico City 11340, Mexico; rcoral@ipn.mx; 6 ROME Psiquiatría Integral, Gregorio Gelati 33, Colonia San Miguel Chapultepec, Miguel Hidalgo, Mexico City 11850, Mexico; 7 Centro Universitario del Norte (CUNORTE), Universidad de Guadalajara, Colotlán 46200, Mexico; edgar.zamora8148@academicos.udg.mx; 8 Instituto Nacional de Rehabilitación, Mexico City 14389, Mexico; bngomez@inr.gob.mx (B.G.D.); gutymen_kriss@yahoo.com.mx (C.G.M.); 9 Departamento Ciclo de Vida, Universidad Autónoma de Guadalajara, Zapopan 45134, Mexico; violetadiazb@gmail.com (M.V.D.B.); sarosas@edu.uag.mx (S.A.R.-M.); 10 Investigadores por México, Secretaría de Ciencia, Humanidades, Tecnología e Innovación (SECIHTI), Mexico City 03940, Mexico; carlos.palma@ipn.mx

**Keywords:** depression, *MTHFR*, functional SNP, folate, Mexican-Mestizos

## Abstract

Background: Major depressive disorder (MDD) arises from genetic and environmental factors. We evaluated functional variation in methylenetetrahydrofolate reductase (*MTHFR*), circulating MTHFR protein, and homocysteine (HCY) in Mexican-Mestizo participants. Methods: We analyzed 1507 individuals across four cohorts: a primary case–control sample (358 DSM-IV-TR MDD cases, 170 psychiatrically screened controls), a community cohort (*n* = 204), and a general population cohort (*n* = 775) for allele-frequency context. We genotyped rs1801133 (C677T) and rs13306560 using TaqMan assays. Serum MTHFR and HCY were measured by ELISA in available subsamples (n reported where applicable). Primary analyses tested association of genotypes with MDD under codominant, allelic, and recessive models; false discovery rate (FDR) correction accounted for multiple genetic models. Exploratory multivariable logistic regression adjusted for sex, age, and adverse childhood experiences. Results: The rs13306560 A allele was rare (~2.6%) and showed no association with MDD. Under a recessive model, the rs1801133 TT genotype was more frequent among cases (33.3%) than controls (23.5%) (χ^2^ *p* = 0.036); this association did not survive FDR correction (q = 0.117) and is reported as hypothesis-generating. In an exploratory adjusted logistic regression (*N* = 446) using the post hoc recessive coding, TT homozygotes had higher odds of MDD versus CC/CT (OR = 1.98, 95% CI 1.23–3.17, *p* = 0.005). Serum MTHFR concentrations were higher in cases than controls (median 1834.8 vs. 1241.9 pg/mL; Mann–Whitney U = 343, *p* = 0.017). Serum MTHFR displayed a borderline positive correlation with HAM-D scores (Spearman r = 0.24, *p* = 0.050). Conclusions: In this Mexican-Mestizo sample, *MTHFR* C677T (rs1801133) homozygosity and elevated circulating MTHFR emerged as preliminary, hypothesis-generating candidates for MDD risk. The genetic association did not meet multiple-testing correction (q = 0.117) and requires independent replication; the serum MTHFR case–control difference survived correction (q = 0.034) but warrants confirmation given the small biomarker subsample. A mechanistic study is needed to clarify causality and clinical relevance.

## 1. Introduction

Major depressive disorder (MDD) is among the most prevalent and disabling psychiatric conditions worldwide, characterized by persistent sadness, anhedonia, neurovegetative dysregulation, and substantial functional impairment (American Psychiatric Association, 2013). Twin and family studies estimate its heritability at between 41 and 49%, with the remaining variance attributable to environmental adversity and gene–environment interactions (Kendler et al., 2018). The impact of environmental stressors varies; among the most consistent moderators are adverse childhood experiences (ACEs)—early-life parental loss, abuse, neglect, and exposure to addiction or domestic violence—which increase lifetime MDD risk, lower the age of onset, and modify clinical trajectory (Dagnino et al., 2025; LeMoult et al., 2020). Developmental stress is therefore considered a critical factor through which inherited biological vulnerability interacts with early-life adversity to shape depressive trajectories across the lifespan.

One of the most biologically plausible pathways linking genetic susceptibility to depression involves folate-dependent one-carbon metabolism and the homocysteine–methylation cycle. Methylenetetrahydrofolate reductase (MTHFR; EC 1.5.1.20) catalyzes the irreversible reduction of 5,10-methylenetetrahydrofolate to 5-methyltetrahydrofolate, the principal methyl donor required for the re-methylation of homocysteine (HCY) to methionine and the subsequent synthesis of S-adenosylmethionine (SAM). The SAM pathway is essential for monoaminergic neurotransmitter synthesis, phospholipid metabolism, epigenetic regulation, and DNA methylation—all processes implicated in the neurobiology of mood disorders (Kurkinen et al., 2021). Dysregulation of this pathway may therefore contribute to depressive phenotypes through impaired methylation capacity, oxidative stress, endothelial dysfunction, and altered neurotransmission (Wan et al., 2018).

**Figure 1:**
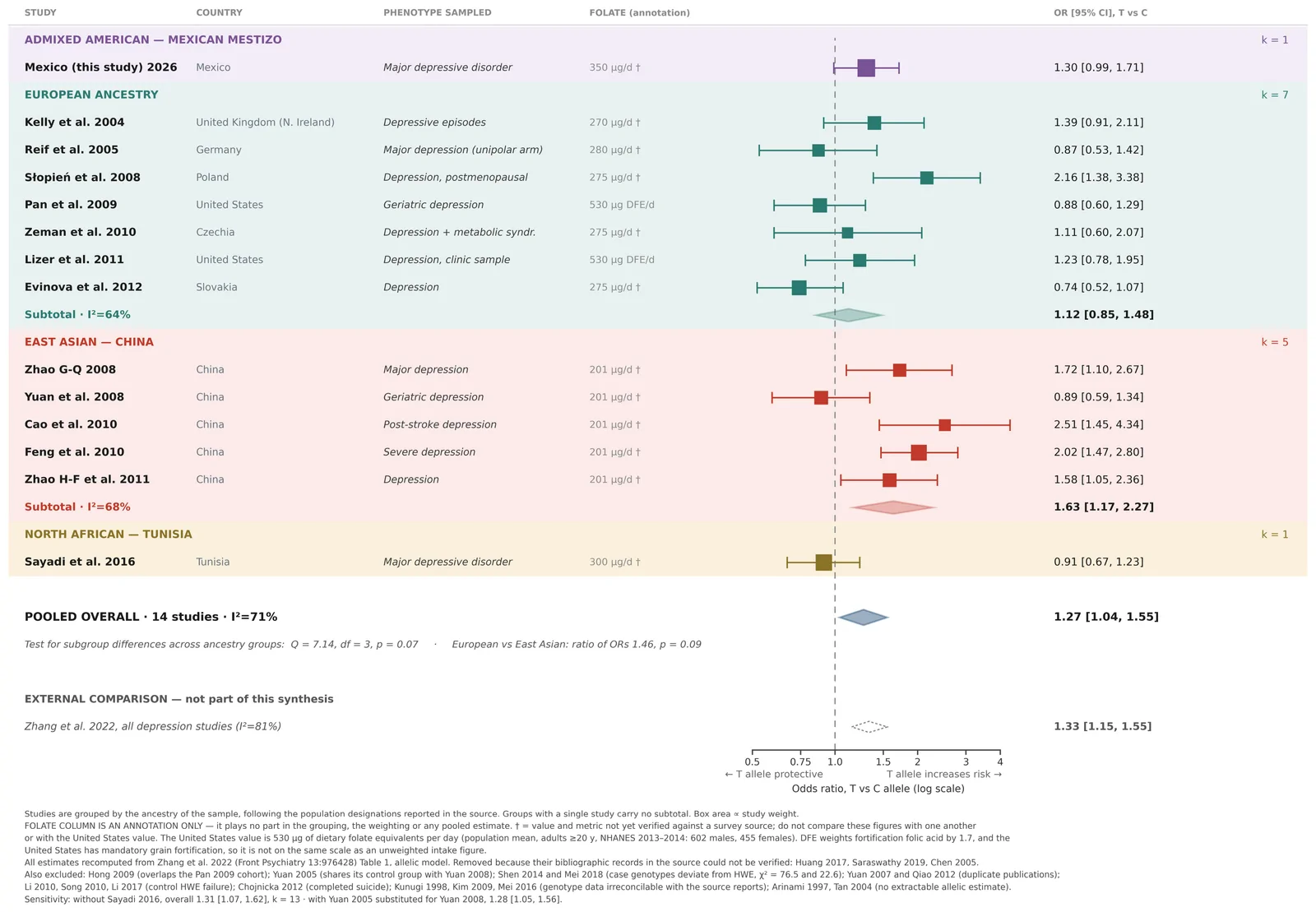
Association between the MTHFR C677T polymorphism and depressive disorders, grouped by ancestry of the sample (allelic model, T vs. C). Random-effects (DerSimonian–Laird) meta-analysis of 14 case–control samples. Squares mark study-specific odds ratios (OR), with area proportional to random-effects weight, and horizontal lines span the 95% confidence interval (CI); the dashed line marks the null (OR = 1); diamonds show pooled estimates, with width equal to the 95% CI; the x-axis is logarithmic. Groups follow the population designations reported in the source compilation, and groups represented by a single study carry no subtotal. The phenotype sampled is printed in italics because the source literature mixes major depressive disorder with geriatric, post-stroke, postmenopausal, and metabolic-comorbid samples rather than a single diagnostic entity. The present Mexican-Mestizo sample gives an OR of 1.30 (95% CI 0.99–1.71). The European-ancestry group (k = 7)—Kelly et al. (2004), Reif et al. (2005), Słopień et al. (2008), Pan et al. (2009), Zeman et al. (2010), Lizer et al. (2011), and Evinova et al. (2012)—pools to 1.12 (0.85–1.48; I^2^ = 64%). The East Asian group (k = 5), all from China—G. Q. Zhao (2008), Yuan et al. (2008), Cao et al. (2010), Feng et al. (2010), and H. F. Zhao et al. (2011)—pools to 1.63 (1.17–2.27; I^2^ = 68%). The single North African sample, Sayadi et al. (2016), gives 0.91 (0.67–1.23). Across all 14 samples the pooled OR is 1.27 (1.04–1.55; I^2^ = 71%). Subgroup differences do not reach conventional significance (Q = 7.14, df = 3, *p* = 0.07; European ancestry vs. East Asian, ratio of ORs 1.46, *p* = 0.09), and because ancestry, country, language of publication, and study size all vary together across these groups, the apparent contrast is a hypothesis rather than an established finding and does not establish a biological difference between populations. The unshaded dashed diamond reproduces the all-depression-studies estimate of Zhang et al. (2022) (OR 1.33, 1.15–1.55; I^2^ = 81%), which used a broader and partly different study set; it is shown for orientation only and is not part of the present synthesis. The folate column is an annotation only: it plays no part in the grouping, the weighting, or any pooled estimate. The United States value is 530 µg dietary folate equivalents (DFE) per day, the population mean for adults aged 20 years and over in NHANES 2013–2014 (602 µg DFE/day in men, 455 in women); because DFE weights folic acid from fortified food by 1.7 and the United States mandates folic acid fortification of enriched grain products, this figure is not on the same scale as an unweighted dietary folate intake. Values marked † have not been verified against a named survey source and their metric is unknown; they should not be compared with one another, nor with the United States value, and no inference about folate should be drawn from their numerical ordering. Genotype counts for the 13 previously published samples were taken from Table 1 of Zhang et al. (2022), and every odds ratio was recomputed from those counts under the allelic model (T = CT + 2·TT; C = 2·CC + CT) with Woolf standard errors. Sensitivity analyses did not change the direction of the overall result: excluding Sayadi et al. (2016) gives 1.31 (1.07–1.62; k = 13), and substituting Yuan et al. (2005) for Yuan et al. (2008) gives 1.28 (1.05–1.56; k = 14). Eighteen of the 31 depression records in the source table were not carried into this figure. Three were removed because their bibliographic records in the source could not be verified: Huang et al. (2017), Saraswathy et al. (2019), and Chen et al. (2005). Fifteen were excluded on methodological grounds: Hong et al. (2009) is an overlapping report of the same geriatric-depression cohort as Pan et al. (2009); Yuan et al. (2005) shares an identical control group with Yuan et al. (2008) and is not an independent sample; Yuan (2007) and Qiao et al. (2012) are duplicate publications of Yuan et al. (2005) and H. F. Zhao et al. (2011), respectively; Shen et al. (2014) and Mei et al. (2018) have case genotype distributions that depart strongly from Hardy–Weinberg equilibrium (χ^2^ = 76.5 and 22.6, 1 df), a check the source meta-analysis applied only to controls; Z. Z. Li et al. (2010), Song (2010), and Y. Li et al. (2017) show control-group Hardy–Weinberg failure already flagged in the source; Chojnicka et al. (2012) reports completed suicide rather than a depressive disorder; Kunugi et al. (1998), Kim et al. (2009), and Mei et al. (2016) have tabulated genotype data that could not be reconciled with the source reports; and Arinami et al. (1997) and Tan et al. (2004) yield no extractable allelic estimate. Furthermore, several studies have reported abnormal folate and HCY levels in individuals with depressive disorders, supporting the hypothesis that both genetic and biochemical alterations in the folate pathway contribute to MDD vulnerability (Brum Moraes et al., 2026). Although associations between rs1801133 and MDD have been observed in multiple populations, the findings remain heterogeneous, likely reflecting ancestry-specific allele frequencies, environmental modifiers, and methodological differences across studies (Contreras-Cubas et al., 2016; Zhang et al., 2022). In contrast, cis-regulatory variation affecting *MTHFR* expression has been far less explored: the *MTHFR* gene is located on chromosome 1p36.22 within the *AGTRAP*–*MTHFR*–*CLCN6* cluster, and the promoter variant rs13306560 has been shown to modulate *MTHFR* transcriptional activity (Pérez-Razo et al., 2015) (mechanistic detail in Section 4). Together, rs13306560 and rs1801133 represent complementary functional variants that affect the folate pathway at different levels—one through altered transcriptional regulation, the other through altered enzyme activity—and their combined contribution to MDD risk has not previously been examined in the Mexican-Mestizo population.

The most studied functional variant in this pathway is the *MTHFR* C677T variant (rs1801133), which causes an alanine-to-valine substitution and produces a heat-labile enzyme with markedly reduced activity (Frosst et al., 1995). T-allele carriers exhibit impaired folate metabolism and elevated plasma HCY levels, particularly when folate intake is low (Crider et al., 2011; Guéant-Rodriguez et al., 2006). Because hyperhomocysteinemia has been consistently linked to depressive symptoms, cognitive impairment, neurotoxicity, and vascular damage, it is regarded as both a biomarker and a potential link in affective disorders. Consistent with this, a large meta-analysis of 81 studies reported that the C677T variant is significantly associated with an increased risk of schizophrenia and major depression, and with bipolar disorder under the recessive model; these associations were stronger in Asian populations (Zhang et al., 2022) (Figure 1).

The Mexican-Mestizo population (most present-day Mexicans) is notably underrepresented in psychiatric genetic studies. Candidate-gene association studies in psychiatric disorders have shown poor replication overall compared with genome-wide approaches, and this record warrants cautious interpretation of any single-cohort finding; *MTHFR* C677T nonetheless remains a defensible candidate because it has a well-characterized functional effect on enzyme activity within a specified biological pathway (folate-dependent one-carbon metabolism) repeatedly, if inconsistently, linked to depression risk, and because it has rarely been studied in Amerindian-admixed populations such as Mexican-Mestizos, where the T allele reaches unusually high frequency. To our knowledge, no previous study has examined the combined contribution of the genetic factors rs13306560 and rs1801133, serum MTHFR enzyme levels, homocysteine concentrations, and ACEs to MDD. In the present study, we evaluated the contribution of two *MTHFR* variants, rs1801133 and rs13306560, along with circulating MTHFR enzyme and HCY levels, to MDD risk. Specifically, we tested the following hypotheses: (1) that the functional variants rs13306560 and rs1801133 are associated with MDD independent of childhood adversity and sociodemographic covariates; and (2) that altered serum levels of MTHFR and homocysteine are associated with depressive state.

## 2. Materials and Methods

### 2.1. Study Design and Participants

#### 2.1.1. Study Design and Overview

This cross-sectional case–control genetic association study was conducted at the Centro Médico Nacional “20 de Noviembre” (ISSSTE), Mexico City. The study included four independently ascertained groups of unrelated adults (total *N* = 1507; group-specific sample sizes reported in Section 2.1). All participants were 18–88 years of age and provided written informed consent. The protocol complied with the Declaration of Helsinki and was approved by the Research and Ethics Committee of the Centro Médico Nacional 20 de Noviembre, ISSSTE (approval number: 382.2014; approved on 17 October 2014).

With the exception of the general-population reference sample, participants self-reported Mexican-Mestizo ancestry, defined a priori as both parents born in Mexico, a surname of Spanish origin, and documented Mexican ancestry through the third generation. This composite criterion is commonly used in Mexican population-genetics studies as a practical proxy for shared continental admixture and was applied to reduce confounding by population stratification given documented heterogeneity of *MTHFR* allele frequencies among Mestizo and Amerindian groups (Contreras-Cubas et al., 2016).

#### 2.1.2. Participant Groups and Ascertainment

##### Major Depressive Disorder Cases (*n* = 358)

Patients were consecutively recruited from the Biomedical Research Division of Centro Médico Nacional 20 de Noviembre between 2014 and 2015. Inclusion required a primary diagnosis of major depressive disorder (MDD) according to DSM-IV-TR criteria (American Psychiatric Association, 2000), independently confirmed by two board-certified psychiatrists; discordant cases were excluded to maximize diagnostic reliability. Depression severity was measured by a trained clinician using the 17-item Spanish Hamilton Depression Rating Scale (HDRS-17). To reduce etiological heterogeneity, participants were excluded for comorbid substance use disorders (except tobacco use disorder), psychotic disorders, manic or hypomanic episodes, schizoaffective disorder, autism spectrum disorder, intellectual disability, neurocognitive disorders, obsessive–compulsive disorder, or trauma- and stressor-related disorders (Table 1).

##### Psychiatrically Screened Major Depressive Disorder Controls (*n* = 170)

Controls were recruited and evaluated by the same clinical team and instruments as the cases. Eligibility matched the cases for the operational definition of Mexican-Mestizo ancestry, age range, and the full set of exclusion criteria. All controls underwent a clinician-administered structured psychiatric interview and were confirmed free of current Axis I psychiatric diagnoses, including MDD, at the time of assessment (Table 1).

##### Community Mental-Health Reference Group (*n* = 204)

This group comprised unrelated individuals who self-identified as Mexican-Mestizo and reported no current psychiatric or neurological diagnosis. Ascertainment relied on clinical history rather than the structured clinician-administered interview used for psychiatrically screened controls (MDD control group); no standardized diagnostic interview was performed. Because this less-intensive screen is less sensitive to subclinical or undiagnosed conditions, we treated this group separately (rather than pooling with psychiatrically screened controls) to avoid potential bias toward the null from nondifferential misclassification (Table 2).

##### General-Population Reference Sample (*n* = 775)

This sample consisted of blood-donor volunteers screened according to standard transfusion eligibility criteria: age 18–65 years, body weight > 50 kg, stable vital signs, and absence of acute or chronic illness. Additional exclusions followed donor-safety guidelines (recent medication use, recent infection, recent surgery or percutaneous procedures, recent alcohol or injected-drug use, pregnancy/lactation, etc.; see Supplementary Table S1 for full list). Ancestry was not formally documented for this group. This cohort was genotyped for rs13306560 only; the rs1801133 (C677T) genotype distribution reported for this group is not this cohort’s own genotyping data but a published reference value from an independent Mexican-Mestizo sample (Contreras-Cubas et al., 2016) (607 of 638 individuals genotyped for this variant), used here for population-level comparison (Table 2). This larger cohort provided greater precision for estimating rare allele frequencies (e.g., rs13306560 minor A allele in *CLCN6*) and served as a reference for circulating homocysteine (HCY) and serum MTHFR measurements (Table 2).

### 2.2. Rationale for Hierarchical Group Comparison

We included multiple comparison groups to evaluate whether genetic associations varied with the stringency of phenotypic ascertainment. Psychiatrically screened controls minimize misclassification but are relatively small; the community reference reflects self-reported Mexican-Mestizo healthy volunteers with a less-intensive screen and may contain undiagnosed or subclinical cases; the large blood-donor cohort provides population-level allele-frequency estimates and reference biomarker distributions. Analyzing these groups separately allows (1) assessment of attenuation from control misclassification, (2) comparison of biomarker distributions across clinical and population samples, and (3) improved precision for low-frequency variants. These complementary groups strengthen both the epidemiological and population-genetic interpretation of findings.

### 2.3. Setting, Ethics, and Consent

Ethics approval, consent procedures, and compliance with the Declaration of Helsinki are described in Section 2.1.1.

### 2.4. Clinical Assessment and Covariates

Trained interviewers collected socio-demographics (age, sex, education, income, marital status, geographic origin) and clinical history. Adverse childhood experiences (ACEs) were assessed using a study-specific structured checklist—rather than a standardized, previously validated ACE instrument—covering parental divorce/separation, familial death, sexual abuse, family addiction, and physical or psychological violence; responses were coded as a single binary present/absent variable, collapsing adversities of differing type and severity into one indicator. Depressive symptom severity was assessed using the 17-item Spanish HDRS. Biomarker collection and laboratory methods are described in Section 2.5.

### 2.5. DNA Isolation and Genotyping

Genomic DNA was extracted from peripheral blood using the dodecyl trimethylammonium bromide/hexadecyltrimethylammonium bromide (DTAB/CTAB) precipitation method. Genotyping of the *MTHFR* rs13306560 and rs1801133 (C677T) variants was performed by real-time PCR using TaqMan hydrolysis probes on a LightCycler 480 II platform (Roche Diagnostics, Basel, Switzerland). The rs13306560 variant was assayed with C__30914969_10, and rs1801133 with C_1202883_20 (Applied Biosystems, Foster City, CA, USA). All reactions followed the manufacturer’s recommended cycling conditions. Genotyping was conducted at the Department of Genomic Medicine, Centro Médico Nacional “20 de Noviembre”, ISSSTE, Mexico City. Hardy–Weinberg equilibrium was tested separately in cases and controls using Pearson’s χ^2^ and Fisher’s exact tests for expected proportions. rs1801133 (*MTHFR*, chromosome 1p36.22; C>T; reference allele C, minor/effect allele T) and rs13306560 (*MTHFR*/*CLCN6* promoter region, chromosome 1p36.22; G>A; reference allele G, minor allele A).

### 2.6. MTHFR and Homocysteine Quantification

Serum concentrations of MTHFR and HCY were quantified in a subgroup of cases and controls using commercially available ELISA kits from CloudClone Corp. (Katy, TX, USA) (MTHFR, catalog No. SEH609Hu) and CUSABIO (Houston, TX, USA) (homocysteine, catalog No. CSBE13814h), following the manufacturers’ instructions. Circulating MTHFR protein concentration, rather than an enzyme-activity assay, was selected as the readout because our a priori hypothesis centered on the promoter variant rs13306560, whose minor A allele is predicted to reduce *MTHFR* transcription through interference with transcription-factor binding; serum protein concentration is therefore the more direct index of this putative expression effect. Notably, too few AA homozygotes were identified to permit a genotype-stratified comparison of serum MTHFR for this variant. Briefly, for both measurements, serum samples were collected using serum separator tubes, allowed to clot for two hours at room temperature, and centrifuged at 1000× *g* for 15 min. The recovered serum was either assayed immediately or stored at −80 °C until analysis, avoiding repeated freeze–thaw cycles. Concentrations were calculated from a four-parameter logistic (4PL) standard curve provided in the kit. The minimum detectable dose of HCY was <0.195 nmol/mL, and for MTHFR the assay range was 23.5–4000 pg/mL. All samples were analyzed in duplicate.

### 2.7. Statistical Analysis

All statistical analyses were performed using jamovi (version 2.6.44) and JASP (version 0.97.0.0). An exploratory data analysis was performed prior to inferential testing. Categorical variables were analyzed using Pearson’s chi-squared test or Fisher’s exact test, as appropriate. Genetic association with case–control status was additionally tested for each variant (rs1801133, rs13306560) under codominant, allelic, and recessive models; false discovery rate (FDR) correction was applied across the three models tested per variant to account for multiple comparisons. Continuous variables were assessed based on their distribution characteristics and the number of comparison groups, applying parametric or non-parametric statistical tests as appropriate; non-normally distributed biomarker concentrations (serum MTHFR and homocysteine) were compared using the Mann–Whitney U test. Because a variance-ratio F-test indicated unequal variances between groups for the serum MTHFR comparison, Welch’s *t*-test was applied alongside the Mann–Whitney U test for confirmation. To evaluate potential selection bias arising from incomplete assay and genotyping coverage, we additionally compared demographic and clinical characteristics (age, sex, Hamilton score, and recruitment source, as applicable) between assayed and non-assayed subgroups for the MTHFR/homocysteine ELISA measurements, and between genotyped and non-genotyped subgroups for each SNP (rs1801133, rs13306560), using chi-squared tests for categorical variables and Welch’s *t*-tests for continuous variables. Variables that showed significant associations in the univariate analyses were subsequently included in binary logistic regression models to assess their independent association with case–control status. Because the recessive model showed the strongest univariate association for rs1801133, this coding (TT vs. CC/CT) was selected post hoc for the exploratory multivariable logistic regression model; this data-driven model-selection step means the resulting adjusted estimates should be interpreted as exploratory and hypothesis-generating rather than confirmatory. Only variables that remained statistically significant after multivariate adjustment were retained in the final models and are presented in this document.

## 3. Results

### 3.1. Sample Characteristics

The case–control analysis included 358 patients with major depressive disorder (MDD) and 170 psychiatrically screened controls. Cases were older than controls (41.8 ± 14.1 vs. 34.7 ± 14.4 years, *p* < 0.001) and included a higher proportion of women (76.8% vs. 48.8%, *p* < 0.001). Differences were also observed in educational attainment (*p* < 0.001) and marital status (*p* = 0.006) (Table 1).

Clinical and psychiatric characteristics differed markedly between groups. A previous depressive episode was reported by 87.4% of cases, and 50.6% were receiving psychiatric medication at the time of assessment. Family history of depression was more common among cases than controls (47.5% vs. 34.1%, *p* = 0.004), whereas family history of psychiatric illness did not differ significantly between groups (*p* = 0.120). Adverse childhood experiences (ACEs) were reported by 61.5% of cases and 38.8% of controls (*p* < 0.001). Mean HAM-D scores were substantially higher among cases than controls (12.8 ± 6.0 vs. 3.1 ± 2.2, *p* < 0.001) (Table 3).

### 3.2. Genotype Distribution and Hardy–Weinberg Equilibrium

Genotype distributions for both genetic variants were consistent with Hardy–Weinberg equilibrium in every group with available genotype counts (Fisher’s exact test: rs1801133—MDD controls *p* = 0.443, MDD cases *p* = 0.540, community reference *p* = 0.855; rs13306560—MDD controls *p* = 1.000, MDD cases *p* = 1.000, community reference *p* = 1.000, general population *p* = 0.179), supporting genotyping reliability. The T allele of rs1801133 was predominant in all groups, consistent with reported frequencies in Mexican-Mestizo populations (Table 2 and Table 3). Linkage disequilibrium between rs1801133 and rs13306560 was computed directly from the observed genotype counts using standard D′ and r^2^ formulas and was negligible in controls (r^2^ = 0.003, D′ = 0.32; *p* = 0.36), consistent with Pérez-Razo et al. (2015). The low r^2^ reflects the low minor-allele frequency of rs13306560 (~2.5%), which constrains the maximum attainable r^2^; thus, the variants were treated as independent.

For rs1801133, codominant (χ^2^ *p* = 0.088) and allelic (*p* = 0.064) contrasts were non-significant. Under a recessive model, TT homozygotes were more frequent in cases than controls (33.3% vs. 23.5%; χ^2^ *p* = 0.036); because this recessive coding was selected after inspecting all three contrasts, we applied Benjamini–Hochberg false-discovery-rate correction across the three rs1801133 genetic models and the rs13306560 carrier-frequency test (m = 4): the recessive-model result did not survive correction (q = 0.117), and this coding is reported as a hypothesis-generating finding rather than a confirmed association; it was nonetheless carried forward into an exploratory multivariable analysis (Section 3.3) given its consistency with prior meta-analytic evidence (Zhang et al., 2022). For rs13306560, the minor A allele was rare (cases: 2.7%; controls: 2.5%), with no AA homozygotes observed. The carrier-frequency difference was non-significant (Fisher’s exact *p* = 1.000; q = 1.000), and the low minor-allele frequency precluded stable estimation of genotypic effects; thus, this variant was excluded from multivariable modeling. Collectively, rs1801133 showed a nominally significant, but not FDR-significant, recessive association with MDD, whereas rs13306560 was not associated. No genotype associations were observed for recurrence status, age at first depressive episode, postmenopausal depression, or current episode status.

### 3.3. Multivariable Association with MDD Case Status

We fitted a binomial logistic regression to jointly evaluate the independent associations of *MTHFR* C677T genotype (recessive coding: TT vs. CC/CT), sex, age, and childhood trauma with MDD case–control status, restricting the analysis to the 446 participants (276 cases, 170 controls) who had non-missing data for case–control status and all four covariates; missing data were assumed to be missing at random, and no imputation was performed. This complete-case sample is smaller than the full case–control sample because genotype data were unavailable for some participants: 276 of 358 cases and all 170 controls had genotype data for rs1801133 (C677T), compared with 346 of 358 cases and 162 of 170 controls for rs13306560 (*CLCN6*); the rs1801133-based sample is used consistently for all regression results reported below. The model fit the data adequately (deviance = 522.9; AIC = 532.9; McFadden’s R^2^ = 0.118), and omnibus likelihood-ratio tests confirmed that all four predictors contributed significantly (Table 4).

In this exploratory adjusted model—built using the post hoc recessive coding described in Section 3.2—each predictor was independently and nominally associated with increased odds of being a case. Carrying the C677T TT genotype nearly doubled the odds of MDD relative to CC/CT carriers (OR = 1.98, 95% CI 1.23–3.17; *p* = 0.005); because this genetic model did not survive FDR correction across the three models tested in Section 3.2, this estimate should be interpreted as hypothesis-generating pending independent replication. Female sex carried roughly threefold higher odds relative to male sex (OR = 2.84, 95% CI 1.85–4.35; *p* < 0.001), and a history of childhood trauma carried nearly threefold higher odds (OR = 2.75, 95% CI 1.81–4.20; *p* < 0.001); older age was associated with a modest per-year increase in odds (OR = 1.03 per year, 95% CI 1.01–1.04; *p* < 0.001). Because education and marital status differed significantly between cases and controls (Table 1) but were not included in the primary model, we re-fitted the model with both added as categorical covariates; the C677T TT association was essentially unchanged (OR = 2.05, 95% CI 1.26–3.32; *p* = 0.004), confirming it is not confounded by either variable, although two education categories with very small, outcome-homogeneous cell counts (technical degree, *n* = 19; no formal schooling, *n* = 1) produced unstable, quasi-separated coefficients that are not further interpreted. Finally, because recruitment was based on available participants rather than a priori power calculations, we conducted a post hoc power analysis for this complete-case sample: power was approximately 83% to detect the observed rs1801133 TT effect (OR = 1.98)—adequate for moderate effects but insufficient for the rare rs13306560 variant, which limited our ability to detect modest associations for that marker.

### 3.4. Serum MTHFR and HCY Concentrations

**Figure 2:**
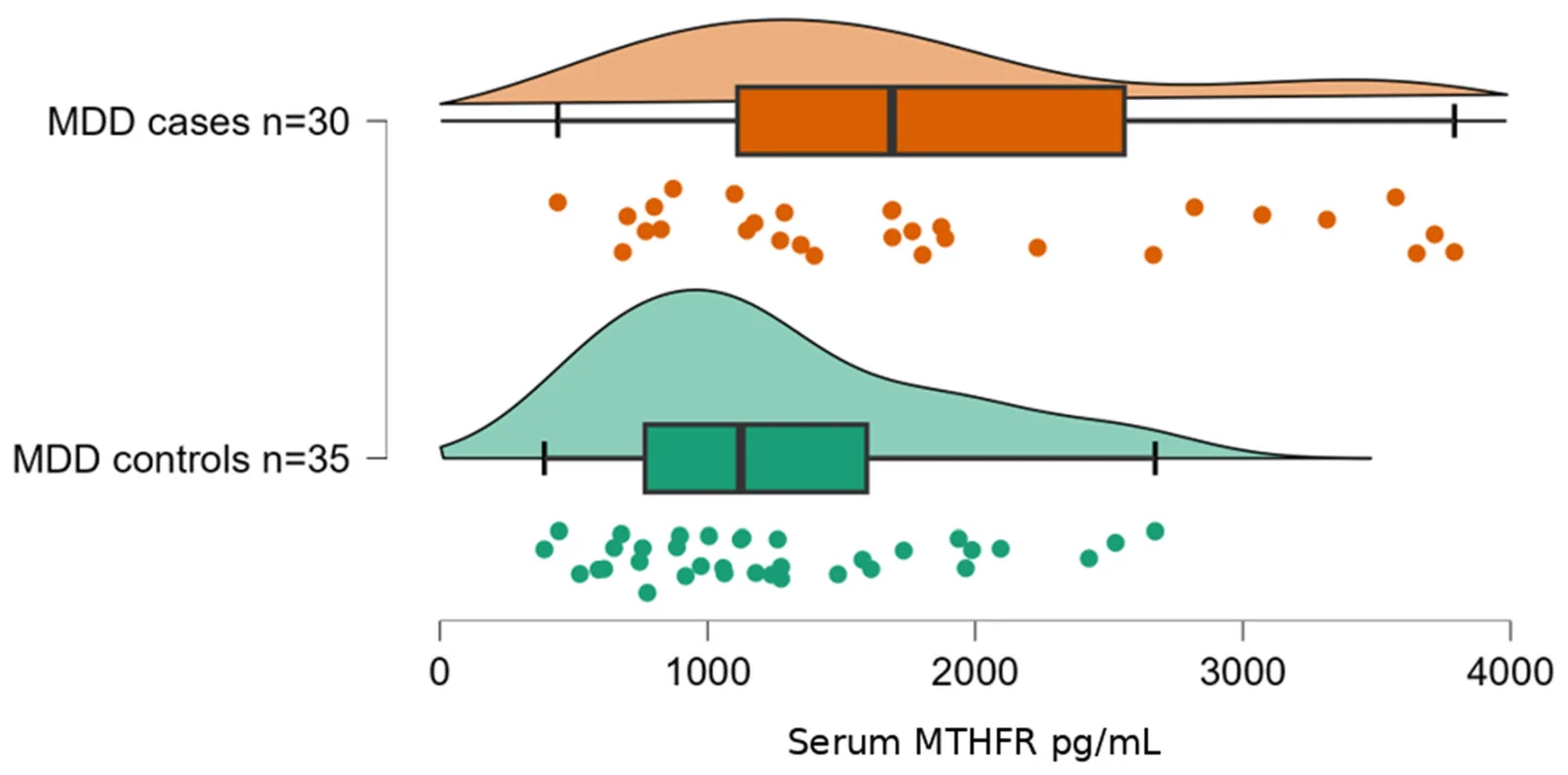
Serum MTHFR concentrations in MDD cases and MDD controls.

Serum MTHFR and HCY concentrations were measured in a small subgroup of MDD cases and controls. Due to non-normal distributions, group differences were assessed using the Mann–Whitney U test. Serum MTHFR concentrations were significantly higher in cases than controls (U = 343, *p* = 0.017; Figure 2), with median values of 1834.8 pg/mL (MDD cases) versus 1241.9 pg/mL (MDD controls). In contrast, HCY concentrations did not differ between groups and showed no associations with depression severity, recurrence, or genotype. Neither rs1801133 nor rs13306560 was associated with serum MTHFR or HCY concentrations. Similarly, among biomarkers, serum MTHFR showed a borderline positive correlation with HAM-D scores (Spearman r = 0.24, *p* = 0.050), whereas HCY was unrelated to age or depression severity.

## 4. Discussion

MDD arises from the interplay of genetic and environmental factors. Our study offers preliminary evidence that functional variation in the folate one-carbon metabolism pathway contributes to MDD risk in a Mexican-Mestizo population—a population markedly underrepresented in psychiatric genetics despite carrying the highest reported *MTHFR* 677T allele frequency in the world. The *MTHFR* rs1801133 (C677T) variant emerges as a candidate genetic vulnerability factor: in an exploratory adjusted model, the homozygous TT genotype was associated with approximately double the odds of MDD, an association that persisted after adjusting for age, sex, and adverse childhood experiences (ACEs). This estimate should be read as hypothesis-generating—it followed a recessive coding selected post hoc after inspecting all three genetic contrasts, and did not survive false-discovery-rate correction (q = 0.117)—but it is a coherent starting point for replication, and the reasons this population is well suited to detect it are themselves informative. The rs13306560 variant, by contrast, was rare and showed no association with disease susceptibility, a null result that turns out to be biologically informative rather than merely negative, as discussed below.

Phenotypically, MDD cases showed a distinct biochemical signature: elevated circulating serum MTHFR protein, with a borderline correlation to depression severity, in a small assayed subgroup (~30 cases, ~35 controls) that warrants cautious interpretation. Serum homocysteine, in contrast, was unchanged between groups and unrelated to genotype or clinical presentation. Read together, these findings point toward a mechanism operating through impaired methyl-donor availability and epigenetic regulation rather than through classical hyperhomocysteinemia—a more specific hypothesis than the field’s default explanation, and one this cohort is reasonably well placed to motivate.

The association is consistent with meta-analytic evidence linking the TT genotype to depression risk, particularly in Asian and other high-T-allele-frequency populations (Zhang et al., 2022) (Figure 1). The C677T substitution produces a thermolabile enzyme with substantially reduced activity, particularly under low-folate conditions (Frosst et al., 1995); a threshold effect of this kind—where a large reduction in catalytic activity is needed before clinical consequences emerge—is consistent with the excess of TT homozygotes we observed among cases. This is where the nutritional context of our cohort becomes genuinely interesting rather than a limitation to explain away. Mexico has mandated folic acid fortification of wheat and nixtamalized corn flours since 2008 under Norma Oficial Mexicana NOM-247-SSA1-2008 (Secretaría de Salud, 2008), so the population carrying the world’s highest 677T frequency is also, by policy, one in which the enzyme’s principal substrate is supplemented at the level of dietary staples. In principle, abundant folate should “rescue” the thermolabile enzyme: the C677T substitution accelerates dissociation of the stabilizing FAD cofactor, and adequate folate or riboflavin can protect against FAD loss, restoring near-wild-type kinetics in vitro (Yamada et al., 2001). Yet the rescue appears incomplete on two counts. First, the policy itself delivers less than it promises: fortification in Mexico has been implemented without direct enforcement, and folic acid content in dietary staples is highly variable, producing unpredictable total folate intake across the population (Orjuela et al., 2019)—consistent with the moderate rather than high folate intake estimate assigned to this cohort in Figure 1. Second, and more fundamentally, even documented repletion does not appear to abolish the variant’s downstream effect: in young Mexican-American women of shared ancestral background, the 677TT genotype produced lower global leukocyte DNA methylation than the CT or CC genotypes even after folate supplementation (Axume et al., 2007). Evolutionarily, the 677T allele reaches its highest worldwide frequency in Mexico, tracking the Amerindian ancestry component of the Mestizo genome (Contreras-Cubas et al., 2016), suggesting this population has carried the variant at high frequency long before any fortification policy existed. Practically, that same high frequency is what gives the present study its statistical purchase: TT homozygosity in 33.3% of cases confers power that lower-frequency cohorts lack, so the adjusted OR of 1.98 likely reflects a more precisely estimated effect in this population rather than an inflated one.

Why might folate availability fail to fully rescue the enzyme where it matters? One possibility is that the brain behaves as a privileged compartment: transport of 5-methyltetrahydrofolate across the blood–brain barrier is rate-limiting, and the brain operates near the ceiling of its methylation capacity, so an enzyme “rescued” peripherally could remain functionally deficient centrally. We did not measure central methyl-donor status or brain methylation, so this is a testable hypothesis motivated by the genotype finding, not a demonstrated mechanism. It connects naturally to reduced S-adenosylmethionine (SAM) availability, which has been linked to global DNA hypomethylation and altered methylation of genes central to mood regulation—including BDNF and genes governing serotonergic and glutamatergic signaling (Gao et al., 2018)—offering a plausible epigenetic bridge between genotype and phenotype that future work could test directly.

Genotype was not the only strong predictor in our model. Childhood trauma was one of the strongest independent predictors of MDD, increasing risk nearly threefold, consistent with the broader literature on early-life adversity and psychiatric outcomes. That both ACEs and *MTHFR* genotype were independently significant raises the possibility of a gene–environment interaction: prospective work has shown that T-allele carriage combined with early-life adversity predicts depression recurrence, with severity scaling with the number and intensity of childhood traumatic events (Lok et al., 2013). Our sample did not permit formal interaction testing, but the simultaneous significance of both variables supports a multifactorial model worth testing directly in a larger sample. Female sex was also independently associated with MDD, consistent with the well-established sex difference in depression prevalence.

Despite the genotype association, serum homocysteine did not differ between cases and controls—a finding worth taking seriously rather than treating as a nuisance result. Circulating homocysteine is shaped by dietary folate, vitamin B12, renal function, smoking, medication, and age, any of which could mask a genotype-dependent effect (Guéant-Rodriguez et al., 2006), and deficits in methylation capacity can occur without measurable changes in serum homocysteine. Any pathogenic effect of the 677TT genotype in this cohort therefore looks more consistent with impaired methyl-donor availability and epigenetic regulation than with homocysteine accumulation—a distinction with real implications for where future mechanistic work should look.

A similar logic of biological specificity helps explain the absence of association between rs13306560 and MDD, which on its face is an unremarkable null result. What makes it worth a closer look is that the same variant is robustly associated with essential hypertension in this same Mexican-Mestizo background (Pérez-Razo et al., 2015). Functional work found that the minor (A) allele reduces *MTHFR* promoter activity by roughly 25% in luciferase assays in human umbilical vein endothelial cells, an effect compounded by CpG methylation of the regulatory region, and that the hypertension association appeared in adults but not children (Pérez-Razo et al., 2015)—a developmental dependence suggesting the phenotype requires a maturing vascular context. *MTHFR* transcription is governed by two promoters with largely non-overlapping activity: a distal promoter active in the neural tube, neural crest, and vascular endothelium, and a proximal promoter that is silent early in development but postnatally drives expression in the hippocampus, cerebellum, and cortex (Pickell et al., 2011), an architecture conserved in the human gene (Pickell et al., 2011; Tran et al., 2002). If rs13306560’s regulatory effect is confined to the vascular promoter, its silence in our psychiatric phenotype would not be a failure of power but exactly what the biology predicts: a vascular compartment variant with no reason to move the needle on a CNS-driven trait. This reframes the null result as a specific, falsifiable prediction—testable directly by comparing rs13306560-dependent *MTHFR* expression in neuronal versus endothelial cell lines—rather than as a dead end. The variant’s low frequency in our sample (~2.5%) limited power for modest effects and is a genuine caveat, but it does not undermine the compartment argument, which rests on the mechanism rather than the sample size.

The direction of the serum MTHFR finding is itself the interesting part of the biomarker data. A higher—not lower—protein concentration in cases is counterintuitive under a simple loss-of-function model. A more compelling reading is compensatory upregulation: the cell increases enzyme abundance to offset reduced per-molecule catalytic efficiency, a response pattern well precedented for other regulatory enzymes under substrate or activity stress. The dissociation between elevated protein and unchanged homocysteine fits this picture and reinforces the genotype-level conclusion that homocysteine accumulation is not the principal mediator here. This hypothesis was not directly tested and would need enzyme-kinetic or expression assays to confirm; given the small assayed subsample, it is best regarded as a promising lead for a dedicated follow-up study rather than a settled mechanism.

Limitations. Several constraints qualify how these findings should be read. This is a candidate-gene association study, a design with a documented history of failing to replicate in larger or genome-wide analyses; the present single-cohort findings should be read with that base rate in mind pending independent replication. The multivariable model adjusted for age, sex, and ACEs; education and marital status differed between cases and controls (Table 1) but were excluded from the primary model, though a sensitivity analysis including both left the C677T association essentially unchanged (OR = 2.05, 95% CI 1.26–3.32; *p* = 0.004; Section 3.3). Smoking status was recorded dichotomously (ever/never) but without quantity, intensity, or duration of exposure, so pack-years could not be derived and residual confounding by smoking dose—which is the dimension most relevant to homocysteine and folate status—cannot be excluded. Other plausible confounders of folate metabolism and depression risk, including BMI, dietary folate and B12 status, nutritional supplementation, socioeconomic status beyond education, medication use, and medical comorbidities, were not measured at all. Notably, individual folate status was not assayed in this cohort; our characterization of the nutritional environment rests on national fortification policy and population-level intake estimates rather than participant-level measurement, and given the documented variability in fortification compliance (Orjuela et al., 2019), individual folate exposure within this cohort is likely heterogeneous. The recessive genetic model was selected post hoc after inspecting codominant (*p* = 0.088), allelic (*p* = 0.064), and recessive (*p* = 0.036) contrasts, and only the recessive coding entered multivariable analysis. We applied Benjamini–Hochberg correction within the family of rs1801133/rs13306560 genetic-model tests (m = 4): the recessive association central to this manuscript (raw *p* = 0.036) corresponds to q = 0.117 and does not survive correction, and is reported throughout as hypothesis-generating rather than confirmed. Within the serum-biomarker family (m = 2), the case–control difference in serum MTHFR survives correction (*p* = 0.017, q = 0.034), but the MTHFR–HAM-D correlation does not (*p* = 0.050, q = 0.050). Broader clinical and demographic comparisons (Table 1) were not corrected, consistent with their descriptive role. Genotyping completion was not uniform across variants and groups, most notably for rs1801133, where data were unavailable for a substantial minority of cases and community-reference participants. This reflects the staged nature of the project: rs13306560 was genotyped first using recently extracted DNA, whereas rs1801133 was genotyped later from archived DNA that had undergone additional freeze–thaw cycles and, in a subset, no longer met quality thresholds for reliable calling. The assay itself (validated TaqMan probes under standardized conditions) was not the source of the discrepancy; samples were excluded on quality and availability grounds alone, independent of participant characteristics. Missingness was nonetheless assumed to be completely at random, an assumption we could not formally test with the available data. The serum MTHFR and homocysteine analyses were restricted to a small subgroup, limiting precision and generalizability. Finally, the cross-sectional design precludes causal inference; the mechanistic interpretations offered above—brain compartmentalization, compensatory upregulation, tissue-specific promoter activity—are hypotheses generated by these findings, not conclusions this study directly tested, and each warrants dedicated biochemical, neuroimaging, or functional follow-up.

## 5. Conclusions

This study provides preliminary, hypothesis-generating evidence that the *MTHFR* C677T TT genotype is associated with MDD risk in Mexican-Mestizo individuals, alongside established risk factors including female sex and childhood trauma. Because the association relied on a post hoc recessive coding and did not survive correction for multiple genetic-model comparisons (q = 0.117), it requires confirmation in an independent, adequately powered sample before being considered established. The unchanged homocysteine levels are consistent with, though do not directly demonstrate, altered methylation capacity or epigenetic regulation as an alternative mechanism to hyperhomocysteinemia—a specific, testable claim rather than a vague one. The rs13306560 null result, read alongside its tissue-restricted regulatory biology, is best treated as a falsifiable prediction for future work rather than a closed question. Studies integrating genomic, epigenomic, nutritional, and environmental data, in adequately powered and preferably genome-wide-informed samples, are needed to establish whether one-carbon metabolism genuinely contributes to depression risk in this population.

## Figures and Tables

**Table 1:** Baseline demographic and clinical characteristics of MDD cases and MDD controls.

Variable	MDD Controls (*n* = 170)	MDD Cases (*n* = 358)
Age, years, mean (SD)	34.7 (14.4)	41.8 (14.1)
Sex, female, *n* (%)	83 (48.8)	275 (76.8)
**Education level, *n* (%)**		
None/illiterate	0 (0.0)	1 (0.3)
Primary	6 (3.5)	22 (6.1)
Secondary	16 (9.4)	34 (9.5)
High school	47 (27.6)	75 (20.9)
Technical degree	0 (0.0)	41 (11.5)
Bachelor’s degree	67 (39.4)	135 (37.7)
Postgraduate	34 (20.0)	50 (14.0)
**Marital status, *n* (%)**		
Single	106 (62.4)	165 (46.1)
Married	45 (26.5)	134 (37.4)
Common-law union	3 (1.8)	19 (5.3)
Separated/divorced	10 (5.9)	27 (7.5)
Widowed	6 (3.5)	13 (3.6)
**Clinical history, *n* (%)**		
Previous depressive episode	0 (0.0)	313 (87.4)
Current medication	41 (24.1)	181 (50.6)
Previous psychiatric treatment	0 (0.0)	182 (50.8)
Family history of depression	58 (34.1)	170 (47.5)
Family history of psychiatric illness	38 (22.4)	104 (29.1)
Childhood trauma (ACE present)	66 (38.8)	220 (61.5)

**Table 2:** Demographic and genetic characteristics of the community mental-health reference group and the general population.

Variable	Community Reference (*n* = 204)	General Population (*n* = 775)
**Demographic characteristics**		
Age, years, mean (SD)	42.9 (13.4)	36.4 (10.9)
Age, years, median (range)	45 (18–65)	35 (18–65)
Sex, female, *n* (%)	154 (75.5)	208 (26.8)
Sex, male, *n* (%)	50 (24.5)	567 (73.2)
**Genetic characteristics, *n* (%)**		
**rs1801133 (677C>T)**	*n* = 120	Contreras-Cubas et al. (2016) (*n* = 607) ^†^
CC	28 (23.3)	139 (22.9)
CT	62 (51.7)	309 (50.9)
TT	30 (25.0)	159 (26.2)
**rs13306560 (G>A)**	*n* = 195	*n* = 775
GG	187 (95.9)	751 (96.9)
AG	8 (4.1)	23 (3.0)
AA	0 (0.0)	1 (0.1)

^†^ This study’s general-population sample was genotyped for rs13306560 only; the rs1801133 (C677T) genotype distribution shown for comparison is a published literature reference value from an independent Mexican-Mestizo sample (Contreras-Cubas et al., 2016).

**Table 3:** Depression severity and genotype distributions in MDD cases and MDD controls.

Variable	MDD Controls (*n* = 170)	MDD Cases (*n* = 358)
**Depression severity**		
HAM-D score, mean (SD)	3.1 (2.2)	12.8 (6.0)
Remission (≤7)	NA	53 (14.8)
Mild (8–16)	0 (0.0)	152 (42.5)
Moderate (17–23)	0 (0.0)	98 (27.4)
Severe (24–30)	0 (0.0)	31 (8.7)
Very severe (≥31)	0 (0.0)	24 (6.7)
**Genetic characteristics, *n* (%)**		
**rs1801133 (677C>T)**	*n* = 170	*n* = 276
CC	39 (22.9)	54 (19.6)
CT	91 (53.5)	130 (47.1)
TT	40 (23.5)	92 (33.3)
**rs13306560 (G>A)**	*n* = 162	*n* = 346
GG	154 (95.1)	327 (94.5)
AG	8 (4.9)	19 (5.5)
AA	0 (0.0)	0 (0.0)

**Table 4:** Binomial logistic regression model predicting MDD case–control status (*N* = 446).

**Panel A. Model fit (*N* = 446; 276 cases, 170 controls)**						
**Deviance**	**AIC**	**McFadden R^2^**				
522.9	532.9	0.118				
**Panel B. Omnibus likelihood-ratio tests**						
**Predictor**	**χ^2^**	**df**	***p***			
*MTHFR* C677T (TT vs. CC/CT)	8.29	1	0.004			
Sex (female vs. male)	23.36	1	<0.001			
Age	14.06	1	<0.001			
Childhood trauma	23.04	1	<0.001			
**Panel C. Model coefficients**						
**Predictor**	***B***	**SE**	***Z***	***p***	**OR**	**95% CI**
Intercept	−1.895	0.362	−5.24	<0.001	0.150	0.074, 0.305
rs1801133 (TT)	0.681	0.241	2.82	0.005	1.975	1.231, 3.170
Sex (female)	1.043	0.218	4.78	<0.001	2.836	1.850, 4.347
Age	0.028	0.008	3.68	<0.001	1.028	1.013, 1.043
Childhood trauma	1.013	0.215	4.71	<0.001	2.754	1.807, 4.197

## Data Availability

Data derived from the present project are included in Supplementary Materials.

## Supplementary Materials

The following supporting information can be downloaded at: https://www.mdpi.com/article/10.3390/bs16091639/s1, Table S1: Full list of exclusion criteria applied to the community-reference group; Table S2: CC Summary Stats; Table S3: MTHFR MannWhitney; Dataset S1: De-identified study dataset.

## Abbreviations

The following abbreviations are used in this manuscript:

ACE	Adverse Childhood Experience
*AGTRAP*	Angiotensin II Receptor Associated Protein
AIC	Akaike Information Criterion
APA	American Psychiatric Association
BDNF	Brain-Derived Neurotrophic Factor
*CLCN6*	Chloride Voltage-Gated Channel 6
CTAB	Hexadecyltrimethylammonium Bromide
CI	Confidence Interval
DSM-IV-TR	Diagnostic and Statistical Manual of Mental Disorders, Fourth Edition, Text Revision
DTAB	Dodecyltrimethylammonium Bromide
ELISA	Enzyme-Linked Immunosorbent Assay
HAM-D	Hamilton Depression Rating Scale
HCY	Homocysteine
HDRS	Hamilton Depression Rating Scale
HDRS-17	17-item Hamilton Depression Rating Scale
HWE	Hardy–Weinberg Equilibrium
MDD	Major Depressive Disorder
MTHFR	Methylenetetrahydrofolate Reductase
OR	Odds Ratio
PCR	Polymerase Chain Reaction
SAM	S-Adenosylmethionine
SD	Standard Deviation
SE	Standard Error
SNP	Single Nucleotide Polymorphism
4PL	Four-Parameter Logistic (curve fitting model)
